# Observational study on the characteristics of COVID-19 transmission dynamics during the first wave of the epidemic in Lusaka, Zambia

**DOI:** 10.11604/pamj.2024.48.42.36724

**Published:** 2024-06-04

**Authors:** Millica Phiri, Tadatsugu Imamura, Patrick Sakubita, Nelia Langa, Moses Mulenga, Marian Matipa Mulenga, George Kapapi, Michael Mwamba, Jane Nalwimba, Deborah Tembo, Kingsley Keembe, Karen Moompizho, Nkomba Kayeyi, William Ngosa, Davie Simwaba, Paul Msanzya Zulu, Fred Kapaya, Raymond Hamoonga, Mazyanga Lucy Mazaba, Nyambe Sinyange, Muzala Kapina, Chie Nagata, Nathan Kapata, Akira Ishiguro, Victor Mukonka

**Affiliations:** 1 Emergency Operation Center, Zambia National Public Health Institute, Lusaka, Zambia,; 2 Incident Management System, Zambia National Public Health Institute, Lusaka, Zambia,; 3 Japan International Cooperation Agency, Tokyo, Japan,; 4 Center for Postgraduate Education and Training, National Center for Child Health and Development, Tokyo, Japan

**Keywords:** COVID-19, contact tracing, secondary transmission, secondary attack rate, overdispersion, super-spreading events, Africa

## Abstract

**Introduction:**

coronavirus disease 2019 (COVID-19) transmission dynamics in the communities of low- and middle-income countries, particularly sub-Saharan African countries, are still not fully understood. This study aimed to determine the characteristics of COVID-19 secondary transmission during the first wave of the epidemic (March-October 2020) in Lusaka, Zambia.

**Methods:**

we conducted an observational study on COVID-19 secondary transmission among residents in Lusaka City, between March 18 and October 30, 2020. We compared the secondary attack rate (SAR) among different environmental settings of contacts and characteristics of primary cases (e.g, demographics, medical conditions) by logistic regression analysis.

**Results:**

out of 1862 confirmed cases of COVID-19, 272 primary cases generated 422 secondary cases through 216 secondary transmission events. More contacts and secondary transmissions were reported in planned residential areas than in unplanned residential areas. Households were the most common environmental settings of secondary transmission, representing 76.4% (165/216) of secondary transmission events. The SAR in households was higher than the overall events. None of the environmental settings or host factors of primary cases showed a statistically significant relationship with SAR.

**Conclusion:**

of the settings considered, households had the highest incidence of secondary transmission during the first wave in Lusaka, Zambia. The smaller proportion of contacts and secondary transmission in unplanned residential areas might have been due to underreporting of cases, given that those areas are reported to be vulnerable to infectious disease outbreaks. Continuous efforts are warranted to establish measures to suppress COVID-19 transmission in those high-risk environments.

## Introduction

Coronavirus disease 2019 (COVID-19), which is caused by severe acute respiratory syndrome virus type 2 (SARS-CoV-2), was first identified in patients with pneumonia of unknown etiology in December 2019 [1,2]. The number of cases reported from different parts of the world increased rapidly, and the World Health Organization characterized the global spread of COVID-19 as a pandemic on March 11, 2020 [3]. In Zambia, the first two cases of COVID-19 were identified with a history of international travel on March 18, 2020 [4]. Zambia experienced its first surge of cases between July and September 2020, (i.e., the first wave), in which the capital city Lusaka was one of the most affected areas in the country [5]. In the effort to contain such a surge of cases in the community, transmission dynamics of COVID-19 have been intensively studied in different parts of the world. It is widely known that COVID-19 spreads in overdispersion patterns, in which 10% of cases become the source of 80% of new infections (e.g., superspreading events (SSE)) [6,7].

It was previously reported that environmental settings associated with the “three Cs” (i.e., crowded places, close-contact settings, confined and enclosed spaces) were likely to generate SSE and transmission of COVID-19 (e.g., workplaces, restaurants, bars, gyms, and churches) [8-11]. Identification of these risk environments for SSE and transmission led to establishment of targeted public health interventions (e.g., temporary closures of restaurants and bars, physical distancing, and wearing masks in houses of worship) to suppress COVID-19 transmission in the community [12-14]. Host factors were also intensively studied as being one of the potential driving forces of COVID-19 transmission [15]. Most of this valuable information was reported from high-income countries (HICs). Transmission dynamics of COVID-19 in low- and middle-income countries (LMICs), including sub-Saharan African countries, are assumed to be different from those in HICs. This is because of the highly varied cultural, socioeconomic, and political backgrounds which potentially affect the behavioral patterns of infected patients, environmental settings of contacts, and timeliness of implementation of mitigation measures [16]. However, numbers of reports describing the transmission dynamics of COVID-19 in LMICs, particularly those in sub-Saharan African countries, are still very limited. In this study, we aim to determine the characteristics of COVID-19 transmission dynamics and risk factors associated with secondary attack rates (SAR) in the community during the first wave of the epidemic in Lusaka, Zambia.

## Methods

**Figure 1:**
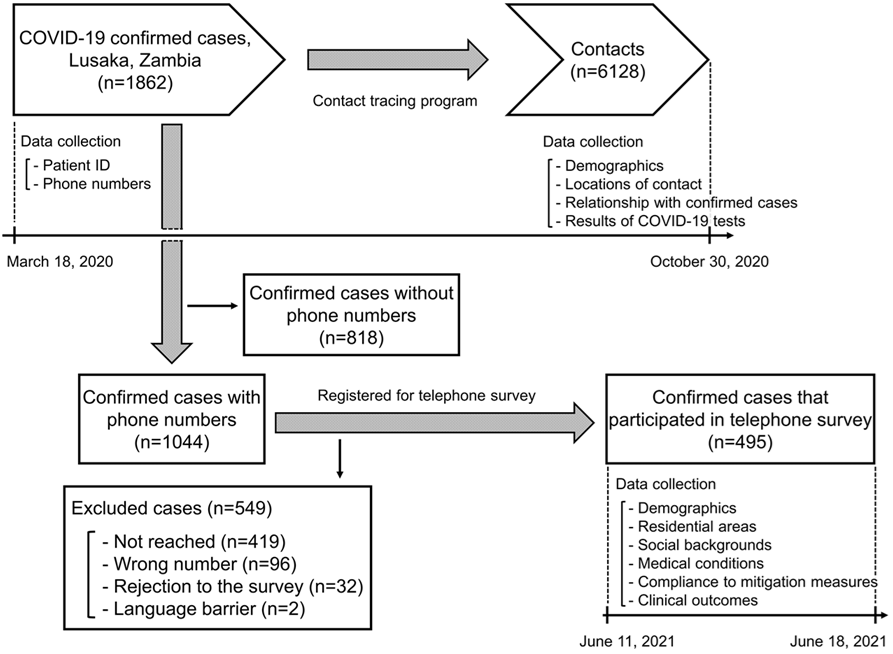
study participants

**Contact tracing program and COVID-19 testing in Lusaka, Zambia:** we conducted a retrospective data analysis of COVID-19 cases and their contacts identified in Lusaka City, the capital city of Zambia, between March 18 and October 30, 2020. The Ministry of Health, Zambia (MoH) through the Zambia National Public Health Institute (ZNPHI) has implemented a nationwide contact tracing program for COVID-19 cases since the first case identification on March 18, 2020 [17,18]. Confirmation of COVID-19 cases was conducted by polymerase chain reaction (PCR) or point-of-care antigen testing using nasopharyngeal swabs that were carried out at COVID-19 testing laboratories designated by MoH and ZNPHI. Following the national guidelines, a person who had contact with a confirmed case within 48 hours before the symptom onset to 14 days after the collection of SARS-CoV-2 positive samples was defined as a contact [19]. If a confirmed case was asymptomatic, the timing of collection of SARS-CoV-2 positive samples was used instead of that of the symptom onset [19]. Contacts were requested to conduct a self-quarantine (i.e., 10 days if they were asymptomatic) and subsequently subjected to COVID-19 testing when they developed symptoms or had suspected infections [19]. Information collected from confirmed cases and their contacts was summarized in the COVID-19 contact tracing database. The COVID-19 contact tracing database contained information including contact tracing identifiers and phone numbers of confirmed cases, geographical locations and environmental settings of the contact, the relationship between the confirmed cases and contacts, and age, gender, and results of COVID-19 testing of contacts (Figure 1).

**Telephone survey for confirmed cases of COVID-19:** to collect background information on confirmed cases of COVID-19, we conducted a telephone survey for previously confirmed cases whose phone numbers were available in the COVID-19 contact tracing database. COVID-19 cases identified through contact tracing efforts of MoH and ZNPHI between March 18 and October 30, 2020, were all eligible for this survey. This survey was not a part of the routine COVID-19 contact follow-ups of MoH and ZNPHI. Trained telephone surveyors called the phone numbers between June 11 and 18, 2021 (Figure 1). We collected information about the confirmed cases including age at the time of COVID-19 diagnosis, gender, residential areas, highest education level, underlying medical conditions, presence of symptoms at the time of diagnosis, attendance to public gatherings and crowded places (i.e., schools, bars and restaurants, malls and markets, hospitals, parties, churches, weddings, funerals) before diagnosis, compliance to wearing masks before diagnosis, compliance to self-quarantine after symptom onset and diagnosis, and clinical outcomes (i.e., hospitalization, intensive care unit (ICU), oxygen therapy, non-invasive positive pressure ventilation (NPPV), high flow nasal cannula (HFNC), ventilation, fatal outcomes). This survey was approved by the Zambia National Health Research Authority (reference number NHRA00001/24/05/2021), and the interview was conducted only after obtaining verbal informed consent from patients in confirmed cases or their family members for participating in the study. If the patients in confirmed cases were younger than 18 years old, verbal informed assent was obtained from them, after which verbal informed consent was obtained from their adult family members or guardians.

**Secondary transmission analysis:** secondary transmission was defined as the identification of new COVID-19 cases among contacts of confirmed cases. Confirmed cases that generated new cases among contacts were defined as primary cases, and the new cases that acquired infection from these primary cases were defined as secondary cases. These definitions were developed from previous studies on secondary transmissions of COVID-19, although the date of symptom onset was not available in our study; the date of confirmation of a positive COVID-19 test was used instead [20,21]. Secondary cases were confirmed to have the same or later dates of confirmation than primary cases. Environmental settings of secondary transmissions were determined based on the information from secondary cases regarding the settings where contacts between primary and secondary cases took place. Secondary transmission which occurred at one environmental setting was counted as one secondary transmission event. If one primary case generated secondary cases in two different environmental settings, the primary case was regarded as generating two secondary transmission events. Secondary attack rates were calculated by dividing the number of secondary cases by the number of contacts for each secondary transmission event.

**Geospatial mapping of COVID-19 cases and contacts:** residential addresses for confirmed cases were collected by the telephone survey. Geographical locations where contacts occurred were collected by physical visits and recordings of the geo-coordinates of the locations were part of the routine contact tracing activities. Digital maps of Lusaka showing the geographical distribution of COVID-19 cases and their contacts were developed using QGIS version 3.10 A Coruña. Township boundaries were accessed through the Zambia Data Hub where they were provided for download by the Ministry of Lands, the Office of Surveyor General. In Zambia, planned residential areas are those officially created by the Government Planning Authority, whereas unplanned residential areas are not. Unplanned residential areas include large numbers of compounds (i.e., informal settlement areas for low-income residents), which are characterized by a high population density, inadequate access to water, sanitation, and hygiene (WASH), and a high incidence of infectious disease outbreaks (e.g., cholera) [22,23]. The geographical distribution of planned and unplanned residential areas was generated and published by Zambia Data Hub [24]. The number of confirmed cases, contacts, and secondary cases were calculated for each of these 94 townships using QGIS.

**Statistical analysis:** it was conducted using R ver.3.5.0 (R Foundation for Statistical Computing, Vienna, Austria). We performed univariate logistic regression analysis to calculate the odds ratio and the 95% confidence interval (95%CI) between SAR and characteristics of cases and contacts. The Wilcoxon rank sum test was performed to compare continuous variables (e.g., numbers of cases and contacts) between planned and unplanned residential areas. A p-value less than 0.05 was considered statistically significant.

**Ethics clearance:** ethics approval for conducting the telephone survey and the use of patients´ data collected as part of the public health response of ZNPHI and MoH for scientific analysis and publication was obtained from the Zambia National Health Research Authority (reference numbers NHRA00001/24/05/2021 and NHRA00002/05/05/2022, respectively).

## Results

**Contact tracing program for COVID-19 in Lusaka, Zambia:** through the contact tracing program in Lusaka City, 1862 confirmed cases were identified in Lusaka, Zambia between March 18 and October 31, 2020. These cases represented 83.0% (1862/2244) of the total laboratory-confirmed cases in Lusaka in the same period. In the contact tracing program, a total of 6128 contacts were identified. The number of contacts per confirmed case was between 1 and 9 for 94.6% (1762/1862), 10-19 for 3.0% (55/1862), 20-29 for 1.2% (22/1862), 30-39 for 0.6% (12/1862), 40-49 for 0.3% (5/1862), and ≥ 50 for 0.3% (6/1862) of the total confirmed cases. Environmental settings of contacts were available for 1418 confirmed cases. These 1418 confirmed cases had contacts in 1584 environmental settings, which led to the identification of 4675 contacts. Environmental settings were unknown (not available) for the remaining 1453 contacts. A total of 166 confirmed cases had contacts in two different environmental settings, whereas others had contacts in only one setting. The largest number of contacts were identified in households (n=3,150), followed by workplaces (n=589), hospitals (n=544), schools (n=177), malls and markets (n=144), police stations (n=49), churches (n=19), and funerals (n=3).

**Telephone survey for confirmed cases of COVID-19:** among 1862 confirmed cases identified in Lusaka, Zambia between March 18 and October 31, 2020, phone numbers were available for 1044 cases (56.1%, 1044/1862). These cases were contacted for eligibility for the telephone survey, among which 495 cases (47.4%, 495/1044) participated in the survey (Figure 1). Among 495 cases that participated in the telephone survey, the most common highest education level attained was higher education (73.3%, 363/495) (Table 1). Underlying medical conditions were reported for 143 cases, among which hypertension was most common (16.2%, 80/495), followed by diabetes (4.2%, 21/495) and human immunodeficiency virus (HIV) infection (3.6%, 18/495) (Table 1). Ninety-seven cases (19.6%, 97/495) were hospitalized, 10 cases (2.0%, 10/495) were admitted to the ICU, and 10 cases (2.0%, 10/495) were ventilated (Table 1). Five cases (1.0%, 5/495) had fatal outcomes.

**Table 1:** characteristics of primary cases of COVID-19

Characteristics	COVID-19 cases (n=495)
Age in years, median (IQR)	38 (31-46)
Male gender, number (%)	241 (48.7)
**Education level, number (%)**	
Primary education	23 (4.6)
Secondary education	95 (19.2)
Higher education	363 (73.3)
No education	3 (0.6)
Unknown	11 (2.2)
**Underlying medical conditions, number (%)**	
Hypertension	80 (16.2)
Diabetes	21 (4.2)
Human immunodeficiency virus	18 (3.6)
Cancer	5 (1.0)
Dialysis	3 (0.6)
Others*	16 (3.2)
Symptomatic at the time of diagnosis, number (%)	293 (59.2)
**Clinical outcome, number (%)**	
Hospitalization	97 (19.6)
Intensive care unit	10 (2.0)
Oxygen therapy	34 (6.9)
Non-invasive positive pressure ventilation or high-flow nasal cannula	17 (3.4)
Ventilation	10 (2.0)
Fatal outcome	5 (1.0)

* Others included anemia, asthma, tonsilitis, bronchiolitis, and hepatitis

**Figure 2:**
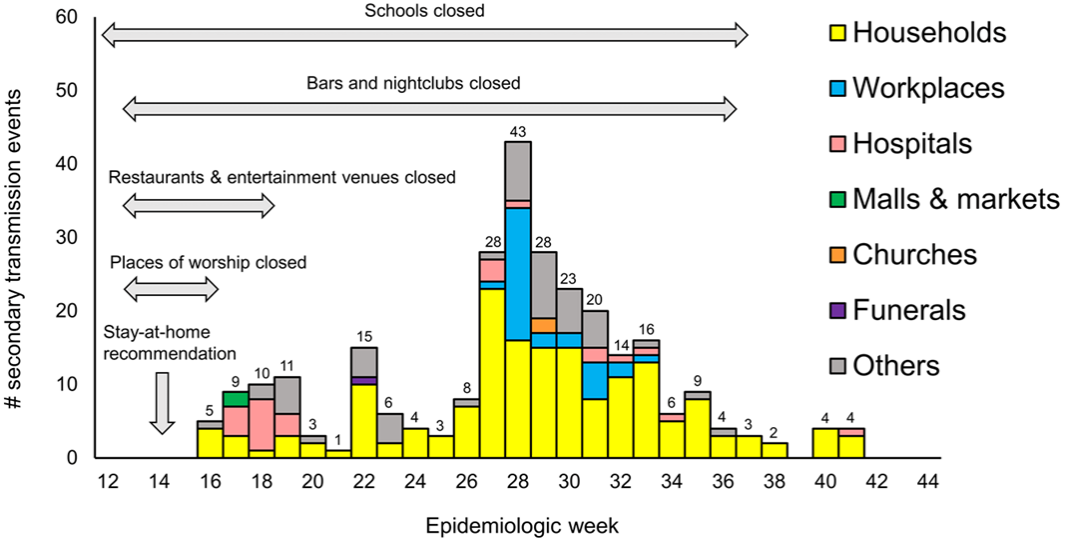
temporal distribution of environmental settings of secondary transmission in Lusaka, Zambia between March 18 and October 31, 2020

**Secondary transmission of COVID-19 in Lusaka:** among 1862 confirmed cases of COVID-19, secondary transmission was generated by 272 primary cases (14.6%, 272/1862). A total of 422 secondary cases were generated, and the number of secondary cases per primary case was 1 for 195 primary cases (71.7%, 195/272), 2-4 for 69 cases (25.4%, 69/272), and ≥ 5 for 8 cases (2.9%, 8/272). Secondary attack rates for overall events was 6.1% (95%CI 5.1-7.1) (Table 2). Environmental settings of secondary transmission were available for 215 primary cases, from which secondary cases were generated in 216 settings. Households were the most common environmental setting for secondary transmission, representing 76.4% (165/216). Secondary attack rates was 6.9% (95%CI 5.6-8.1) for secondary transmission events in households, and 6.8% (95%CI 2.4-8.5) in schools, both of which were higher than SAR for overall events (Table 2). Secondary attack rates were highest in funerals (33.3%, 95%CI 0.8-90.6) and churches (10.5%, 95%CI 1.3-33.1), although there was only 1 secondary transmission event in each of these settings and the numbers of contacts were small (3 and 19, respectively). Secondary attack rates was lowest in police stations with 2.0% (95%CI 0.1-10.9), followed by 2.1% (95%CI 0.4-6.0) in malls and markets and 4.6% (95%CI 5.1-7.0) in hospitals (Table 2). Among 8 primary cases that generated ≥ 5 secondary cases, environmental settings for transmission were available for 3. These 3 transmission events took place in households (1 primary case and 7 secondary cases), workplaces (1 primary case and 7 secondary cases), and schools (1 primary case and 8 secondary cases). After the first identification of cases in Zambia on March 18 (epidemiologic week 12), increased numbers of secondary cases were generated in hospitals between weeks 17 and 19 (Figure 2). During the peak of the epidemic between weeks 27 and 33, there were variations in the environmental settings of secondary transmission (e.g., households, hospitals, workplaces, churches) (Figure 2). After week 34, settings of secondary transmission were limited mainly to households (Figure 2).

**Factors associated with numbers of contacts and secondary attack rates:** among these factors, SAR showed a negative correlation when contacts occurred in malls and markets (OR 0.4, 95%CI 0.1-0.8) (Table 2). None of the factors including demographics, education levels, underlying medical conditions, compliance to mitigation measures, and clinical outcomes, had a statistically significant relationship with SAR (Table 3, Table 4, Table 5).

**Table 2:** associations between environmental settings and secondary attack rates of COVID-19 cases in Lusaka, Zambia

Factors	Total number of confirmed cases	Total number of contacts	Total number of secondary cases	SAR (95%CI)†	OR (95%CI)††
Overall	1862	6128	422	6.1 (5.1-7.1)	-
Settings of contact					
Households	1052	3150	225	6.9 (5.6-8.1)	Reference
Churches	3	19	2	10.5 (1.3-33.1)	1.4 (0.2-4.9)
Funerals	2	3	1	33.3 (0.8-90.6)	4.5 (0.2-35.1)
Hospitals	248	544	28	4.6 (5.1-7.0)	1.0 (0.7-1.4)
Malls and markets	23	144	3	2.1 (0.4-6.0)	0.4 (0.1-8.3)
Police stations	38	49	1	2.0 (0.1-10.9)	0.5 (0.1-1.6)
Schools	45	177	12	6.8 (3.6-11.5)	0.8 (0.4-1.4)
Workplaces	173	589	35	5.4 (2.4-8.5)	0.9 (0.6-1.2)

† SAR: secondary attack rate; 95%CI: 95% confidence interval; ††OR: odds ratio; 95%CI: confidence interval (95%CI or binomial 95%CI when appropriate); OR was calculated by logistic regression.

**Table 3:** associations between social background and secondary attack rates of COVID-19 cases in Lusaka, Zambia

Factors	Total number of confirmed cases	Total number of contacts	Total number of secondary cases	SAR (95%CI)†	OR (95%CI)††
**Age category of primary cases**					
0-19	18	55	4	8.1 (3.6-41.4)	Reference
20-59	366	1456	113	8.0 (5.8-10.2)	1.1 (0.4-3.6)
> 60	21	100	1	0.8 (0.1-23.8)	0.3 (0.0-1.4)
Gender of primary cases					
Female	213	698	59	7.9 (5.0-10.8)	Reference
Male	241	1138	82	7.4 (4.8-10.0)	0.9 (0.6-1.3)
**Highest education level of primary cases**					
Higher	343	1476	110	7.0 (5.0-9.1)	Reference
Secondary	78	247	20	8.3 (3.0-13.5)	1.0 (0.6-1.5)
Primary	22	82	8	9.8 (4.3-18.3)	1.0 (0.4-1.9)
No education	2	2	0	NA	NA

† SAR: secondary attack rate; 95%CI: 95% confidence interval; †† OR, odds ratio; 95%CI, confidence interval (95%CI or binomial 95%CI when appropriate); OR was calculated by logistic regression.

**Table 4:** associations between compliance to public health measures and secondary attack rates of COVID-19 cases in Lusaka, Zambia

Factors	Total number of confirmed cases	Total number of contacts	Total number of secondary cases	SAR (95%CI)†	OR (95%CI)††
**Primary cases attended public gatherings or crowded places within 14 days before diagnosis**					
No	123	564	44	10.3 (6.0-14.6)	Reference
Yes	326	1275	89	6.7 (4.6-8.8)	0.7 (0.5-1.1)
**Compliance of primary cases to wearing masks before diagnosis**					
No	66	299	24	6.1 (1.3-10.9)	Reference
Yes	342	1405	118	8.5 (6.2-10.8)	1.8 (1.0-3.6)
**Compliance of primary cases to self-quarantine after onset and diagnosis**					
No	376	1571	121	7.7 (5.6-9.8)	Reference
Yes	79	309	29	7.3 (2.9-11.6)	1.3 (0.8-1.9)

† SAR: secondary attack rate; 95%CI, 95% confidence interval; †† OR: odds ratio; 95% CI: confidence interval (95%CI or binomial 95% CI when appropriate): OR was calculated by logistic regression.

**Table 5:** associations between clinical background and secondary attack rates of COVID-19 cases in Lusaka, Zambia

Factors	Total number of confirmed cases	Total number of contacts	Total number of secondary cases	SAR (95%CI)†	OR (95%CI)††
**The medical condition of primary cases**					
No conditions	284	1074	82	7.9 (5.4-10.3)	Reference
Hypertension	79	339	22	4.0 (0.8-7.3)	1.0 (0.2-1.9)
Diabetes	21	108	6	5.6 (2.1-11.7)	1.3 (0.4-3.3)
Human immunodeficiency virus	17	84	6	7.1 (2.7-14.9)	0.7 (0.2-1.6)
Cancer	5	13	0	NA	NA
Dialysis	4	16	1	6.3 (0.2-30.2)	NA
**Symptomatic primary cases at the time of diagnosis**					
No	178	651	45	6.1 (5.1-7.1)	Reference
Yes	274	1147	93	8.4 (5.7-11.0)	1.1 (0.8-1.7)
**Disease severity of primary cases**					
Home therapy*	334	1212	97	8.1 (5.8-10.4)	Reference
Hospitalization (w/o oxygen therapy)	53	256	17	8.3 (2.0-14.5)	0.8 (0.5-1.4)
Conventional oxygen therapy	11	46	4	11.4 (2.2-51.8)	1.3 (0.4-3.3)
Intensive care**	26	112	11	2.4 (1.0-25.1)	1.5 (0.7-2.7)
Fatal outcome	6	55	4	3.2 (0.4-77.7)	1.3 (0.2-4.5)

* Patients who had been diagnosed with COVID-19, but were not hospitalized; ** Intensive care: patients who were hospitalized in intensive care units and/or treated with non-invasive positive pressure ventilation (NPPV), high flow nasal cannula (HFNC), or ventilation; † SAR, secondary attack rate; 95%CI: 95% confidence interval; †† OR: odds ratio; 95%CI: confidence interval (95% CI or binomial 95%CI when appropriate); OR was calculated by logistic regression.

**Geospatial distribution of COVID-19 cases and contacts:** among 94 townships in Lusaka, locations, where contacts and secondary transmission occurred, were distributed among 85 townships, including 62 categorized as planned residential areas and 23 categorized as unplanned residential areas (Annex 1(A, B, C, D). The median numbers of contacts per 105 population in each township were significantly larger in those categorized as planned residential areas than in those categorized as unplanned residential areas (Annex 1C, Table 6). Similarly, median numbers of secondary cases per 105 population in each township were also significantly larger in those categorized as planned residential areas than in those categorized as unplanned residential areas (Annex 1D, Table 6). We looked further into the residential areas of 435 confirmed cases (23.4%, 435/1862) for which residential addresses were available. These 435 cases had residents in 61 townships, including 46 planned residential areas and 15 unplanned residential areas (Annex 1B). Among these 61 townships, median (IQR) numbers of confirmed cases per 105 population were significantly larger in planned residential areas than in unplanned residential areas (Annex 1B, Table 6).

**Table 6:** distribution of confirmed and secondary cases and contacts in planned and unplanned residential areas in Lusaka, Zambia

Variables	Planned residential areas (n=65)	Unplanned residential areas (n=29)	P-value**
**Residential areas of confirmed cases**			
Numbers of confirmed cases per 105 population*†	22.2 (0.0-53.5)	1.6 (0.0-6.5)	<0.001
**Locations of contacts and secondary transmission**			
Numbers of contacts per 105 population*	223.0 (91.6-394.3)	30.5 (9.9-46.2)	<0.001
Numbers of secondary cases per 105 population*	775.2 (0.0-1587.3)	81.8 (0.0-238.2)	0.005
**Ratios among contacts, secondary cases, and confirmed cases**			
Numbers of contacts per confirmed case*†	5.2 (3.4-13.7)	9.0 (2.8-31.9)	0.488
Numbers of secondary cases per contact*†	0.1 (0.0-0.1)	0.1 (0.0-0.1)	0.790
Numbers of secondary cases per confirmed case*†	0.3 (0.0-1.0)	0.3 (0.1-1.7)	0.595

* Median (IQR) numbers are indicated; † Residential areas were available for 435 confirmed cases (23.4%, 435/1862); ** The Wilcoxon rank sum test was performed to compare the number of cases and contacts, and ratio (i.e., contacts/confirmed cases, secondary cases/confirmed cases) between planned (n=65) and unplanned residential areas (n=29); A p-value less than 0.05 was considered as statistically significant.

## Discussion

We reported the characteristics of COVID-19 transmission dynamics and factors associated with SAR in the community during the first wave of the epidemic in Lusaka, Zambia. During the first wave of the epidemic, the majority of secondary transmissions were generated in households. Although the early phase of community transmission was comprised of those in a variety of settings, such as hospitals, schools, malls, and markets, it was contained mainly in households as the epidemic evolved. These changes were assumed to be the results of public health interventions implemented by MoH and ZNPHI, including enhanced infection prevention and control (IPC) measures in hospitals, and mitigation measures imposed for the general population (e.g., stay-at-home, school closures). Our findings were in line with a previous report from the United Kingdom that described a high proportion of hospital transmission in the early phase of community transmission, potentially due to inadequate preparedness in testing capacities, IPC skills, and medical equipment, such as personal protective equipment (PPE), during those periods [25].

Among these environmental settings, SAR in households was higher than that for the overall events, although it did not reach a significant difference. High SAR in households might have been due to multiple factors, including prolonged and frequent contact and reduced compliance to IPC measures (e.g., physical distancing, wearing masks) in those settings. Similar results have been reported from other countries, which is indicative of the difficulties in suppressing COVID-19 transmission in households [26-29]. Funerals have been considered to be a high-risk environmental setting as close physical contacts often occur [30]. Although we observed a high SAR for funerals, the number of contacts were too small to draw any conclusions. Conversely, SAR in environmental settings including hospitals and police stations was smaller than that for the overall events. Strict IPC/mitigation measures applied to those settings might have been effective in reducing the number of transmissions [31,32].

We also reviewed our contact tracing program during the first wave in Lusaka. The contact tracing program was conducted for over 80% of the total confirmed cases identified during the period, which was comparable to or higher than those in HICs during the early phase of epidemics [33]. In the contact tracing program, a larger proportion of contacts and secondary transmissions were reported in planned residential areas than in unplanned residential areas. In addition, the number of confirmed cases involving residents of planned residential areas was larger than those of unplanned residential areas. Based on the assumption that chances of acquiring infections were similar in any geographical area, or even larger in highly populated unplanned residential areas, such differences might have been attributable to reduced health-seeking behaviors and underreporting of COVID-19 cases in unplanned residential areas. Targeted public health interventions specific for individuals in those areas (e.g., enhanced risk communication strategies, active case finding) are required to suppress COVID-19 transmission in Lusaka, Zambia.

In Lusaka, COVID-19 transmission occurred in overdispersion patterns, as only less than 15% of confirmed cases generated secondary transmission [6]. This finding is consistent with previous reports from different parts of the world, which suggests that this is a unique characteristic of COVID-19 transmission irrespective of the geographical region [34]. We could identify a very limited number of SSE, despite the vigorous contact tracing activities that covered over 80% of the total confirmed cases during the period. We cannot rule out the potential effects of the limited testing capacity, contact tracing skills, and registration systems during the first wave. However, it also could have been affected by the design of the contact tracing program. In contrast to the widely adopted contact tracing strategy focusing on individuals who had contact with confirmed cases 48 hours before diagnosis/onset (i.e., “prospective” contact tracing), an alternative strategy called “backward/retrospective” contact tracing, which focuses on individuals who had contact with confirmed cases after 14 days before diagnosis/onset, has been currently adopted in several countries to increase the capacity to identify SSE [35-37]. Such an alternative approach might need to be considered to enhance our capacity to contain COVID-19 transmission due to SSE in Zambia.

**Limitations:** limitations of our study include the relatively small number of confirmed cases with background information available and potential inclusion bias due to this, reconstruction of pairs of primary and secondary cases based on the date of confirmation, reduced number of confirmed cases in unplanned residential areas, and potential recall bias among participants in the telephone survey. Furthermore, effects of variant strains (e.g., delta variant) and vaccination were not taken into account, as this study was conducted for cases identified before the appearance of the delta variant and vaccine deployment in Zambia. A recent study from the Netherlands reported that COVID-19 vaccination led to an approximately 20% reduction in primary cases generating household transmission [38]. The effects of vaccination on suppressing COVID-19 transmission in households and communities of Lusaka need to be further evaluated.

## Conclusion

We reported the characteristics of COVID-19 transmission dynamics and risk factors for high SAR during the first wave in Lusaka, Zambia. Households were the major risk environmental settings for secondary transmission under strict mitigation measures. Continuous efforts are required to establish measures to suppress COVID-19 transmission in those high-risk environments, as well as to better identify SSE and cases in unplanned residential areas in Lusaka, Zambia.

### What is known about this topic

- *COVID-19 spreads in over-dispersion patterns in the community;*
- *COVID-19 transmission is likely to occur in environmental settings with the “three Cs (crowded places, close-contact settings, confined and enclosed spaces)” in high-income countries*.

### What this study adds

- *Households were the most common environmental settings for COVID-19 secondary transmission during the “first wave” in Lusaka, Zambia;*
- *A smaller proportion of contacts and secondary transmissions were reported in unplanned residential areas (i.e., informal settlement areas for low-income residents) than in other areas, potentially due to the reduced health-seeking behaviors and underreporting of COVID-19 cases in those areas*.

## Annex

**Annex 1**: geographical distribution of confirmed cases, contacts, and secondary cases of COVID-19 in Lusaka, Zambia between March 18 and October 31, 2020: A) types of residential areas; B) # confirmed cases/105 population; C) # contacts/105 population; D) # secondary cases/105 population (PDF - 109 Kb)
